# Advances in 3D Printing for Tissue Engineering

**DOI:** 10.3390/ma14123149

**Published:** 2021-06-08

**Authors:** Angelika Zaszczyńska, Maryla Moczulska-Heljak, Arkadiusz Gradys, Paweł Sajkiewicz

**Affiliations:** Institute of Fundamental Technological Research, Polish Academy of Sciences, Pawinskiego 5b St., 02-106 Warsaw, Poland; azasz@ippt.pan.pl (A.Z.); mheljak@ippt.pan.pl (M.M.-H.); argrad@ippt.pan.pl (A.G.)

**Keywords:** tissue engineering, 3D printing, biomaterials

## Abstract

Tissue engineering (TE) scaffolds have enormous significance for the possibility of regeneration of complex tissue structures or even whole organs. Three-dimensional (3D) printing techniques allow fabricating TE scaffolds, having an extremely complex structure, in a repeatable and precise manner. Moreover, they enable the easy application of computer-assisted methods to TE scaffold design. The latest additive manufacturing techniques open up opportunities not otherwise available. This study aimed to summarize the state-of-art field of 3D printing techniques in applications for tissue engineering with a focus on the latest advancements. The following topics are discussed: systematics of the available 3D printing techniques applied for TE scaffold fabrication; overview of 3D printable biomaterials and advancements in 3D-printing-assisted tissue engineering.

## 1. Introduction

Recent progress in the 3D printing method stems from the regenerative ability of the human body. It was reported that there were about 31 million Americans who suffered from body defects [1]. Every year, there is globally an increasing number of patients suffering from various types of body defects caused by injuries and degenerative processes of various origin [2,3]. Critical defects require support for the growth of the cells [4]. Native regeneration of the human body is limited by multiple elements such as availability of the growth hormones or by functionality of the defected tissue. For many years, the standard medical treatment in such cases has been autologous transplantation (less frequently, allologous) or implantation of an endoprosthesis imitating the lost organ. The above-mentioned methods allow to restore the full or partial function of the lost organ (tissue defect); however, it should be noted that they are characterized by many disadvantages affecting the comfort of the patient’s life. Hence, the idea of developing methods supporting the full regeneration of tissue defects was born, which are based on laboratory cell cultures collectively referred to as tissue engineering (TE).

Tissue engineering belongs to a group of relatively new fields of human activity. It combines elements of biology, medicine, material engineering, and mechanics. The basic aim of tissue engineering is to develop methods supporting the regeneration of damaged tissues and organs, especially those so far considered to be non-regenerative. Examples of such tissue and organ damage are provided by everyday clinical practice. These are usually critical defects of bone, skin, or nerve tissue. The most common cause of such defects is various types of trauma, with the second most common being those resulting directly from tumor activity or those resulting from resection of tumor sites. Typically, the regenerated tissue (cell culture) is initially cultured in vitro (in a bioreactor), and then the partially regenerated tissue is implanted in situ at the site of the defect. To ensure an even distribution of the cells in the defect space, so-called TE scaffolds are used, which are porous structures that provide an appropriate substrate for the cultured cells and, at the same time, allow free access to nutrients and drainage of cell metabolism products. An equally important task of tissue scaffolds is to take over the mechanical function of the damaged tissue (organ). For this reason, they should be characterized by appropriate stiffness. It is also expected that the implanted scaffold will be fully resorbed by the time the tissue defect is fully regenerated. To meet this requirement, scaffolds are most often fabricated from biodegradable polymers, either natural, such as chitosan or cellulose, or synthetic (polycaprolactone (PCL), polylactide (PLA), etc.). It is not uncommon to use ceramic materials (β-TCP, hydroxyapatite) in a polymeric matrix to improve the biocompatibility of the material used. The designed TE scaffolds must meet many different requirements (in practice, often contradictory). It also turns out that how the scaffold performs its function is determined by factors of various nature, ranging from purely biological to mechanical.

There are numerous methods of TE scaffolds’ fabrication. Amongst them, one can mention a few conventional methods, such as the solvent casting method, phase separation, or electrospinning, which enable limited control over the scaffold geometry. Additionally, they are characterized by poor repeatability. The above limitations do not apply to the additive manufacturing (AM) methods, commonly known as 3D printing methods. Additionally, 3D printing methods enable easy application of computer-assisted methods of TE scaffold design. Presently, there are a multitude of 3D printing techniques applied for TE purposes. They enable fabrication of TE scaffolds made of different types of materials including polyesters, ceramics, metals, or hydrogels.

Generally, an incredible advantage of 3D printing is the possibility of the fabrication of complex structures, unprofitable to manufacture using injection molding methods [5]. Furthermore, 3D printers have been improved for extremely high resolution, which fosters their use in tissue engineering. There are documented attempts of the adaptation of industrial printers to make them usable for printing scaffolds for tissue engineering. Nowadays, 3D printing methods enable fabrication of TE constructs used for the regeneration of different types of tissues, such as skin [6], cartilage [7], and vascular networks [8], as well as whole organs [9].

This review summarizes limitations and general principles of the most extensively used additive manufacturing technologies, including extrusion-based as well as jetting systems. Thus, current methods of printing and printable materials will be discussed. Additionally, the article highlights advanced scaffold fabrication methods for tissue engineering applications.

## 2. Scaffolds for Tissue Engineering

Daily, by average, 13 people die due to a long waiting time for organ transplantation [10]. There exists also a problematic issue related to tissue compatibility. In such a situation, tissue engineering may offer various unique methods of scaffold formation, where the tissue compatibility issue may be easily overcome. The idea and the goal is to deliver a functional compatible organ using the patient’s own cells. However, such a process may be a highly complex task as there exist numerous factors related to the organism’s physiology, such as culturing many cell types [11]. In general, scaffolds are essential for the creation of graft structures. TE scaffolds are a substratum for cells’ migration/differentiation and the creation of new regenerated tissue. Thus, properties of the materials, especially chemical and physical, as well as the architecture and morphology, are crucial for cell proliferation and viability [12,13]. Moreover, successful repair of the defects sometimes requires reconstruction of different types of coexisting tissues, such as bones, glands, muscles, vessels, ligaments, nerves, and cartilage. The scaffolds’ morphology and architecture are crucial at various levels: macro, micro, and nano. At the macro level, the architecture is related to the scaffold size and shape from the perspective of the size and shape of the defect, which are essential for the contact and interactions between the scaffold and the native tissues, matrix-cell interactions, and nutrients’ transport [14]. At the micro level, it is characterized by scaffold porosity, pore shape, or pore spatial distribution, each of which is responsible for general scaffold permeability. At the nano level, the morphology is related to the fiber surface characteristics, which are supposed to be responsible for cells’ differentiation and proliferation [15].

The most critical factors in 3D printing scaffolds are the type of fabrication method and the choice of a biomaterial. Biomaterials interact with biological systems and can be classified by various criteria such as biodegradability, physical and chemical composition, or the application of certain modifications [16]. The choice of a biomaterial is connected with the character of the damaged tissue. Favored materials are usually biodegradable and piezoelectric biomaterials. The main groups of these materials consist of polymers (synthetic and natural), ceramics, and composites. Ceramic scaffolds are preferred in orthodontic applications; composite scaffolds have applications in dental tissue engineering, whereas polymers are used in soft tissue engineering [17].

### 2.1. Different TE Strategies

Generally, two distinct strategies are used in TE to treat tissue defects using tissue scaffolds [18]. In each, the fabricated scaffold is seeded with cells (sometimes cells are embedded in a scaffold matrix), followed by cell culture in a bioreactor, after which the scaffold filled with the newly formed tissue is implanted into the defect site. The difference lies in the choice of the moment of implantation. In the first of the strategies, fully matured and remodeled tissue is implanted in the defect site. In this case, the scaffold should be completely degraded and metabolized before the moment of implantation. In the second strategy, a scaffold filled with not fully matured tissue is implanted. Depending on the strategy chosen, the implanted scaffold should be characterized by different degradation (erosion) kinetics.

**Figure 1:**
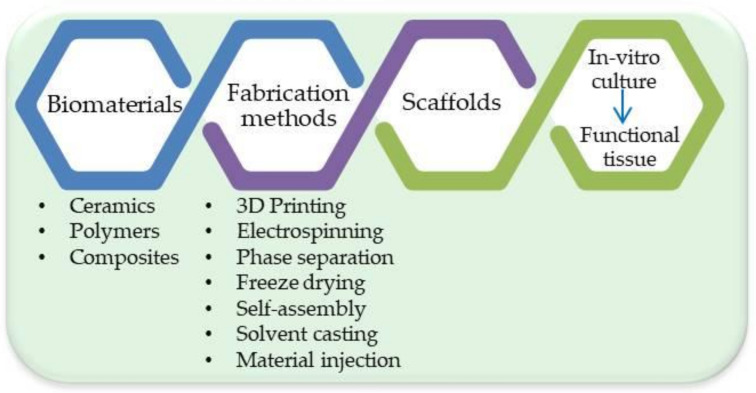
Tissue Engineering process.

TE scaffolds’ fabrication is followed usually by adequate surface modifications in order to achieve the desired structure/properties from the cells’ perspective. Various hormones or growth factors are usually added during the cell culture. Figure 1 shows the process of creating the tissue engineering product.

### 2.2. Conventional TE Scaffold Fabrication Techniques vs. 3D Printing Techniques

**Figure 2:**
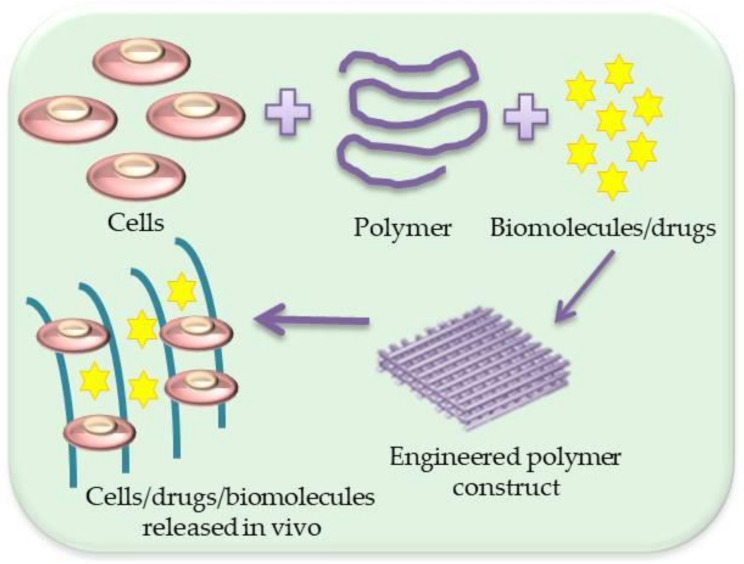
Schematic illustration of scaffold with cells/drugs or biomolecules’ formation.

There are various methods of scaffold formation allowing them to meet the requirements in various specific applications. In addition, many biomaterials are constantly improved for more effective use in tissue engineering. A schematic illustration is shown in Figure 2.

**Figure 3:**
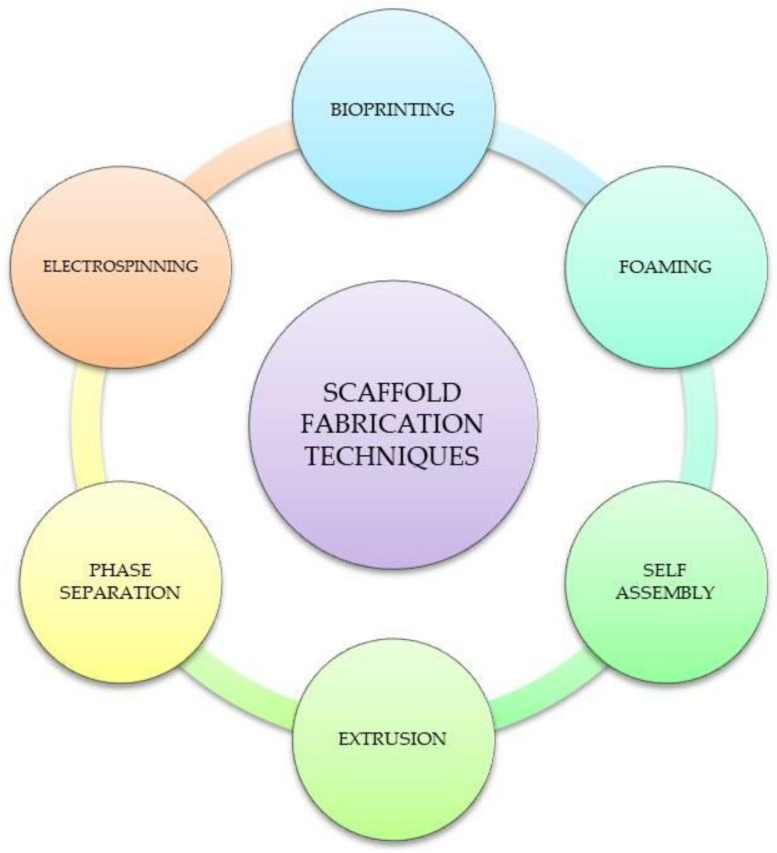
Scaffolds’ fabrication techniques.

The mostly used scaffold fabrication methods include: electrospinning, additive manufacturing, phase separation, solution casting, foaming, extrusion, and self assembly [19]. In order to limit some disadvantages of the methods, a combination of them is often used, which sometimes leads to very interesting and promising effects [20]. Figure 3 shows various techniques to fabricate three-dimensional scaffolds while some of them are described further.

**Figure 4:**
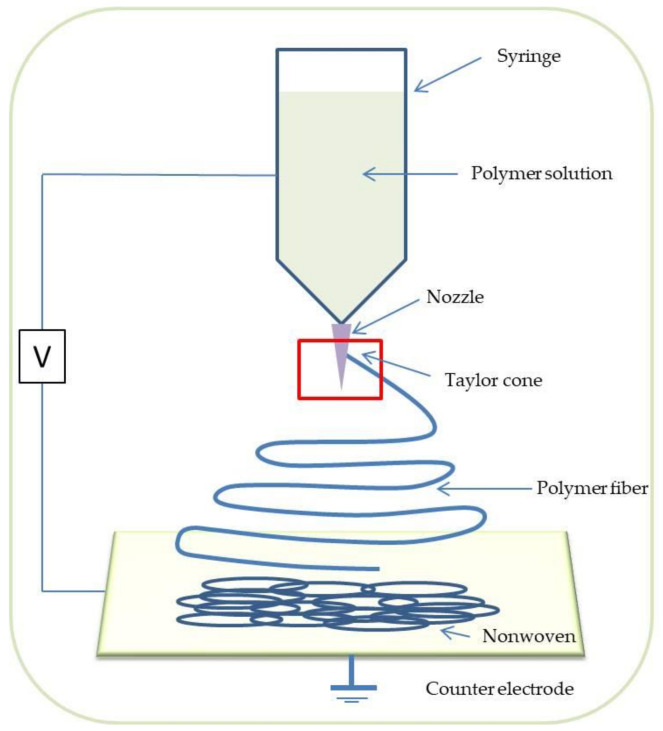
Scheme illustration of electrospinning technique.

One of the most popular processes for scaffold formation is the electrospinning technique (Figure 4). The spinneret filled with electroconductive polymer, usually a solution, is connected to a high electric potential (several to tens kV) at low current. The polymer is spun in the form of fibers, while solvent is evaporating on the way between a spinneret and a collector. A collector is electrically grounded or at a low counter potential and may be stationary or rotating. The resulting scaffold consists of a micron, submicron, or nanofibrous architecture, either random or aligned, depending on the collector type and mode used. This method of scaffold fabrication allows the formation of fibrous nonwovens with morphology and architecture mimicking the fibrous structure of the extracellular matrix (ECM) which is crucial from the perspective of cells. In this process, a large number of various polymers and solvents can be used, both natural, such as gelatin, chitosan, collagen, etc., and synthetic, such as polycaprolactone (PCL) [21], polyvinylidene fluoride (PVDF) [22], poly(3-hydroxybutyrate-co-3-hydroxyvalerate) (PHBV) [23], poly(methyl methacrylate) (PMMA) [24], etc. By connecting different types of materials, hybrid materials can be developed, particularly as a mixture of synthetic and natural polymers. Although electrospinning is a relatively simple process from the instrumental perspective, it is quite complex when analyzing physical phenomena on the process between the moment of jet formation and collection of fibers on the collector. The electrostatic field between the liquid and collector results in a cone-shaped polymer solution to flow out (s.c. Taylor cone). Then, the polymer jet is ejected from the Taylor cone when the electric field exceeds the polymer liquid’s surface tension, followed by various instabilities including bending instability of the jet due to repulsion of the electric charges on the jet surface. By modifying some parameters of the electrospinning process, of the materials, and of external conditions, such as the solution flow rate, spinneret-collector distance, the rotational speed of the collector, voltage, polymer concentration, polymer molecular weight, humidity, and temperature, the morphology and architecture of the scaffold can be dramatically changed according to the desired application [25].

Phase separation is another traditional method to produce complex and high-porosity three-dimensional scaffolds. There are various modifications of this method, which are generally based on two processes, namely, liquid-solid and liquid-liquid phase separation. They are technically implemented by using either thermally or non-solvent-induced processes. In the first case, separation is obtained by reducing polymer solubility through a change in the solution temperature resulting in polymer precipitation. In the second case, phase separation is obtained by immersion of a polymer solution in a non-solvent (for the polymer) bath in order to leach away the polymer solvent (wet phase inversion method). There are additional techniques of porous scaffolds’ formation by phase separation techniques based on highly compressed gases or supercritical fluids. The phase separation method has been developed and improved over the years and used as a manufacturing method to form porous polymer membranes. The resulting morphology of phase-separated scaffolds is extremely sensitive to the process parameters, so desired parameters of three-dimensional scaffolds can be achieved by adjusting various process and materials’ parameters [26].

Mixing an organic solvent with polymers, adding granules or spheres as porogens, and casting this solution to the mold, followed by the extraction of porogens, is known as the solvent casting/particulate leaching method (SCPL). It is possible to control the final pore size by the size, content, and distribution of porogens. The solvent evaporates, and the porogen is removed by dissolving, leaving behind a porous structure. Solvent scaffolds can be used, for instance, for cardiac tissue engineering applications due to the uniform distribution of endothelial cells [27]. This technique allows preparation of structures with regular porosity, but with a quite limited thickness. A summary of these methods is given in Table 1.

Tissue engineering requires fundamental systemic understanding of the human organism including cellular differentiation and proliferation [31,32,33,34]. To summarize, the prerequisites of TE scaffolds (not only those 3D printed) are extremely challenging and manifold. They include: he material for TE scaffold fabrication should be biocompatible (that is, scaffolds cannot cause any cytotoxicity or immune response); scaffolds should be easy to sterilize to prevent infections. Moreover, mechanical properties should be enough for patients’ regular life and activity [18].

## 3. 3D Printing of Tissue Engineering Scaffolds

### 3.1. Overall Characteristics of 3D Printing Techniques

Since the emergence of the concept of using tissue engineering products in reconstructive medicine, many methods of producing TE scaffolds have been developed, starting from the simplest ones, such as the method of sugar- or salt-crystal-leaching from a solid structure, to the most advanced ones, which include rapid prototyping (RP) and rapid manufacturing (RM) methods. The methods of rapid manufacturing are currently a very dynamically developing field. Practically on an ongoing basis, modifications are made to existing methods; new methods and devices are created, and the RM industry is now created both by scientific institutions and commercial manufacturers of hardware and software. Unfortunately, the dynamism of industry development makes it difficult to systematize existing methods. Many of the common names of RM methods are registered trademarks, which means that often even several manufacturers produce very similar devices using different names for virtually the same manufacturing method used by the devices. These names come into common use at the same time, which creates a lot of confusion. One needs to be aware of the fact that terms such as additive manufacturing, rapid prototyping/manufacturing, solid free-form fabrication, as well as 3D printing, are essentially synonymous. In the remainder of this paper, we have chosen to use the term 3D printing. It is a relatively new method of the fabrication of TE scaffolds with controlled architecture. Despite the fact that there are many various 3D printing techniques, including stereolithography, bioprinting, inkjet printing, fused deposition modelling (FDM), PED (Precision Extruding Deposition), laser beam melting, polyjet, electron beam melting, digital laser printing (DLP), and selective laser sintering (SLS) [35], the common feature of all mentioned methods is the general principle of material deposition layer-by-layer until the final product is created [36].

Thus, the 3D TE scaffold is fabricated by the successive addition of consecutive 2D layers of a material. Additive manufacturing has numerous advantages, such as the ability to create complex structures and the possibility of the application of the Computer-Aided Design (CAD) methods. It enables the use of various types of biomaterials [37]. Using living cells and biodegradable polymers allows for the development of methods and novel strategies to create complex tissues and, possibly in the future, whole organs [38]. A 3D-printed TE scaffold can be designed using patient-specific data. The CAD method allows for the precise designing of the 3D organ or its missing part. Selected features of living organs, such as porosity or vasculature, may be taken into account in the CAD 3D model. Due to these remarkable advantages, 3D printing is gaining significant interest in regenerative medicine and tissue engineering [39].

In 3D printing, techniques may be distinguished into two categories—binder 3D printing and direct 3D printing.

**Figure 5:**
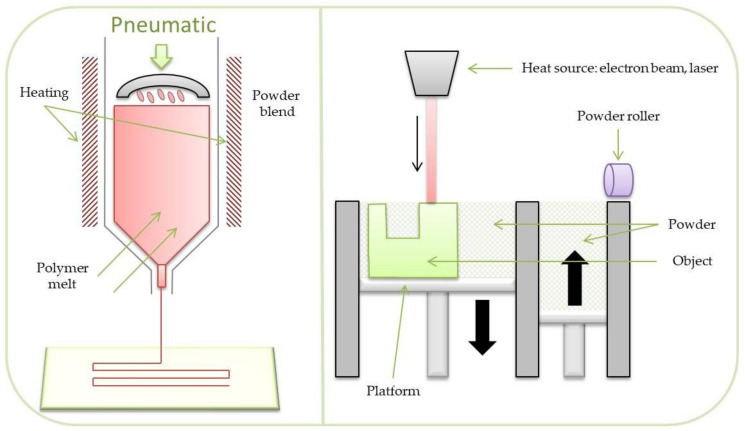
Scheme illustration of direct 3D printing technique (left) and “drop on powder technique” (right).

The former is also called the “drop on powder technique” (Figure 5) [40]. Objects are made by an inkjet liquid printing binder solution on a powder base [41,42,43]. The process starts by spreading the powder layer on the building platform. Positioning software prints the pattern using a deposition of droplets on the layer with powder. Next, the building platform, powder, and part are lowered, and the next layer can be applied. Then, the powder is removed, and one can observe the printed part. The disadvantages of the method include relatively low resolution and problems with printhead reliability. A small nozzle can have better quality but is more prone to clogging. As an advantage, the fabrication of complicated scaffolds with internal channels is feasible because the surrounding powder supports objects.

In the case of direct 3D printing, which is shown in Figure 5, the nozzle of a 3D printer moves back and forth dispensing waxes or plastic polymers, which solidify to form consecutive layers of the fabricated 3D object.

### 3.2. 3D Printing Techniques Applicable to TE

Below, the most-known 3D printing techniques, which are applicable to TE, are listed.

#### 3.2.1. Bioprinting

This method allows for the fabrication of soft 3D tissue scaffolds combining biomaterials, living cells, as well as growth factors. It enables the fabrication of biomedical parts that maximally imitate natural tissue characteristics. Generally, 3D bioprinting utilizes the layer-by-layer deposition of materials known as bioinks to create tissue-like structures. There are four main categories of 3D bioprinting: inkjet bioprinting, laser-assisted bioprinting, extrusion bioprinting, and stereolithography [44].

#### 3.2.2. Inkjet Bioprinting

**Figure 6:**
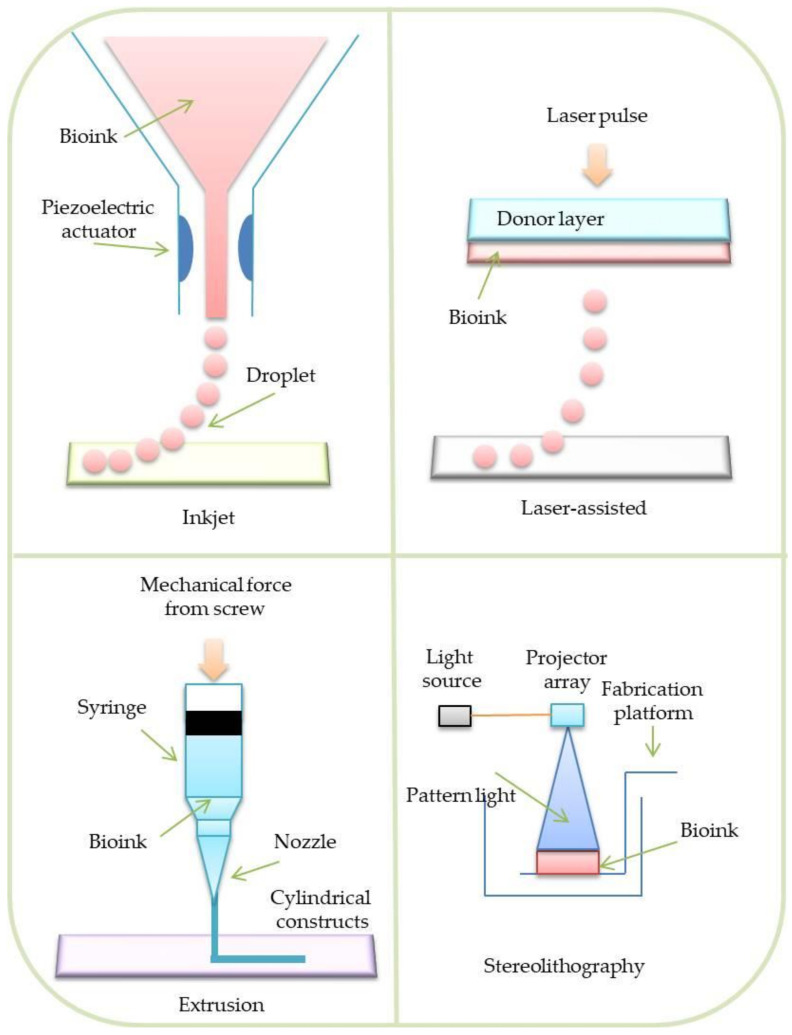
Four main categories of bioprinting.

In this type of bioprinting method, a mixture of living cells and a bioink is stored in a chamber joined with the printhead [45]. During the process, the piezoelectric transducer deforms the printhead. Spatially defined droplets establish tissue constructs (Figure 6). The main advantage of the method is its low cost and high cell viability [46]. Nevertheless, this method is limited by numerous problems, such as printhead clogging, uneven distribution of the cells, and inability to print viscous materials. Due to these problems, inkjet bioprinting has received less consideration by researchers in recent years [47].

#### 3.2.3. Laser-Assisted Bioprinting

Typical laser-assisted bioprinting (LAB) involves specialized layers, such as a bioink layer, an energy-absorbing layer, a donor (quartz/glass), and a collecting layer, to form structures [48]. During the process, a laser beam is focused on the energy-absorbing layer. Next, this layer vaporizes and creates an air bubble between the bioink and donor layers. The formation of a bubble ejects the desired amount of the bioink on the collecting layer. A tissue structure is created in a droplet-by-droplet manner (Figure 6) [49]. LAB is feasible for use with high cell density and viscous materials. Additionally, it has been reported that the method is characterized by high cell viability (95%) and resolves the clogging issues. Nevertheless, LAB is an expensive process, which generates a very high cost with large-scale projects. Therefore, only a few printer prototypes were created. [50,51].

#### 3.2.4. Extrusion Bioprinting

The extrusion bioprinting technique is based on liquid extrusion (paste, solution) from a pressurized syringe through a needle to a solution with controlled density. The materials are extruded in a form of long strands or dots to create complex structures [52]. The printing process can be conducted at room temperature and used to print natural biomaterials, especially hydrogels (Figure 6) [53].

#### 3.2.5. Stereolithography

Stereolithography (SLA) is the first developed method of rapid prototyping expanded in the late 1980s [54]. Stereolithography rasters use a laser beam to control the polymerization process of bioinks in a 2D layer. After the deposition of each layer of a material, curing follows. During the curing process, a photosensitive hydrogel is subjected to UV or visible light. When a given layer is polymerized, the process is repeated, overlapping the previous layer, up to the moment when the whole scaffold is completed. This method allows the use of the following hydrogel materials (Figure 6) [55]: Polyethylene glycol diacrylate (PEGDA) and gelatin methacryloyl (GelMA) [56]; photo-initiators can be also added [57,58]. The adjustment of various polymerization process parameters, including light energy and intensity, speed of printing, layer thickness, and exposure time, enables the achievement of a high-quality (including resolution) product [59,60,61,62,63,64]. Nevertheless, compared to the other methods, the SLA process is relatively time consuming, which makes the process feasible for small-detailed objects.

### 3.3. Fused Deposition Modeling and the Other Microfiber Extrusion Methods

**Figure 7:**
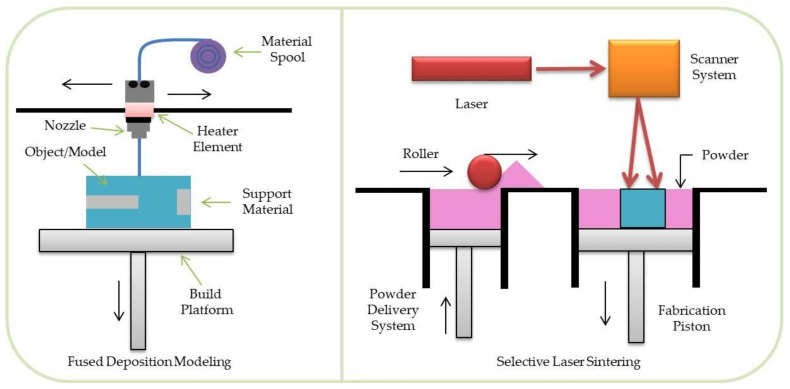
Scheme illustration of FDM (left) and SLS (right) process.

In the fused deposition modelling (FDM) (Figure 7, left) technique, a coiled polymer filament is heated up and extruded through a nozzle on the platform. When the polymer contacts with the platform, it solidifies [65]. The main limitations of using FDM printers in TE include spatial resolution and possible thermal degradation of the polymeric material. FDM enables the use of thermoresponsive polymers such as polycaprolactone (PCL), polylactide (PLA), or polyglycolide (PGA). One of the criteria for selecting a material suitable for FDM is its high thermal stability, which many aliphatic polyesters, unfortunately, do not have. Thermal degradation of plastics is a particularly noticeable problem in the case of devices processing polymer granules (the original FDM method is less exposed to the negative effects of this phenomenon). The polymer heated for a long time loses its viscosity suitable for the proper course of the manufacturing process. In other 3D printing techniques belonging to the polymer microfiber extrusion group, the method of the material supplying may be different. In the case of the precision extruding deposition (PED), the material is supplied in the form of polymer granules, which are thermally plasticized and extruded under pressure through a nozzle. The described group of methods has been successfully used in the fabrication of TE scaffolds for many years. Thanks to the methods based on microfiber extrusion, tissue scaffolds with a strictly planned fibrous structure can be obtained. The disadvantages of the method include the fact that, due to a too-high polymer processing temperature, it is not possible to produce scaffolds with biomolecules or living cells incorporated into the fiber structure.

### 3.4. Selective Laser Sintering

In the method, the polymeric powder particles are heated up slightly above the polymer glass transition temperature by a laser beam [66]. This leads to partial melting of the particles [67], during which molecular diffusion on the particles’ surface takes place, which leads to particles’ fusion. After fabrication of each object layer, the building platform is lowered, a new layer of powder particles is spread on the top and connected with the previous layer (Figure 7, right).

### 3.5. Melt-Spinning

Melt electrospinning (MES) is a relatively new 3D TE scaffold fabrication technique, being the alternative to conventional solution electrospinning (SES) known for disadvantages related to toxic polymeric solutions [68]. Residues of solvents, e.g., chloroform, DMSO (dimethyl sulfoxide), DMF (dimethyloformamid), that can be used by SES may be harmful to living cells seeded on the scaffold. SES limitations were overcome by the use of the molten polymer instead of the polymer solution. To be jetted in an electric field, the molten polymer should be characterized by a suitable viscosity. The molten polymer would be collected by a rotating drum; however, implementation of the numerical control (NC) enables the precise deposition of fiber in X, Y axes. The mentioned approach makes the MES another class of 3D printing techniques [69]. Recent works on the melt electrospinning report that this technique allows for depositing continuous fibers characterized by a diameter less than 1 micrometer, which is comparable to the classic solution of electrospinning [70].

Summarizing information about 3D printing methods are listed in Table 2.

## 4. Design Strategies of 3D Printed Scaffolds

### 4.1. Idea of Computer-Aided Tissue Engineering

Modern tissue engineering probably could not exist without the use of various types of computer-aided methods; however, it was not until numerically controlled 3D printers were introduced in TE that all the advantages of computer-aided TE scaffold design became fully available. They are present at almost all possible stages of creating the so-called tissue engineering product. This chapter aims to characterize selected computer-aided design methods and determine the role they play in the process of tissue scaffold design and fabrication by 3D printing techniques. Generally, the role of computer-aided design in tissue engineering is so important that the term CATE (Computer-Aided Tissue Engineering) has emerged and been used in the literature for some time now [89,90].

**Figure 8:**
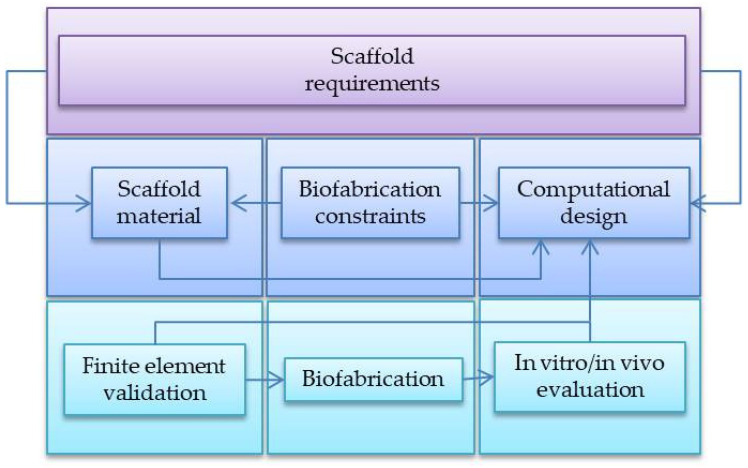
General idea of CATE (based on [90]).

Figure 8 shows a block diagram that, in simplified terms, describes the operation of the CATE system. The blocks in the diagram symbolize the individual modules of the system. In brief, the task of the CATE system is to generate (based on the defect geometry and a set of appropriately selected criteria) a tissue scaffold design in a form comprehensible for numerically controlled manufacturing devices such as 3D printers.

### 4.2. TE Scaffold CAD Geometry Development

Scaffold geometry can be generated from the start to the finish using CAD software. Such a model is usually described by a set of solid virtual objects with surfaces that precisely define its shape. However, it should be kept in mind that the geometry of the scaffold is a representation of the tissue defect, which usually has an irregular shape. In such a case, a more adequate way to acquire the geometry of the designed scaffold would be to use the reverse engineering techniques enabling one to define precisely the defect shape based on the results of medical imaging by means of computed tomography (CT) or magnetic resonance (MRI). The result of the CT examination is a series of cross-sectional images of the examined object called tomograms. The tomograms usually require filtering of all kinds of noise and artifacts typical for this method. The next stage is the binarization of grayscale tomograms. Based on the series of binarized tomograms, one can create a CAD model of the designed scaffold using commercial or free software (Materialise MIMICS, 3DSlicer, InVesalius). The CAD model is usually saved in one of the neutral formats used by additive manufacturing systems. Probably the most common format used in additive manufacturing systems is the Standard Tessellation Language (STL). It was originally developed for stereolithography but later became popular in other additive manufacturing methods. In the STL format, the shape of an object is approximated by a mesh of triangles; hence, the contents of an STL file are the x, y, z coordinates of each vertex and a vector normal to the triangle plane. In addition to STL, there are other less common geometry storage formats such as SLC (a format containing consecutive sections described by polylines), HGPL (HP Graphical Language), and CLI (Common Layer Interface).

### 4.3. Computer-Assisted Optimization of TE Scaffolds

The optimal tissue scaffold should be characterized by many, often contradictory, features. In turn, the number of design variables describing the tissue scaffold structure is so large that a trial-and-error design usually becomes tedious and inefficient, given that experimental evaluation of the design variants involves lengthy and expensive in vitro and in vivo testing. Design variables that directly affect the quality of the designed scaffold include the mechanical properties of the material used, porosity, scaffold stiffness (dependent on the material and scaffold structure), biological activity, and chemical activity of the chosen material. Several theories have emerged as to what the optimal scaffold should be, but for a long time, there was a lack of proven methods for assisting the design of tissue scaffolds. It was not until the mid-1990s that the first attempts to use computer-aided design methods appeared. Until recently, the use of computer methods in tissue engineering was usually limited to the computer-aided design of TE scaffold geometry or the use of in silico models (mainly based on the finite element method (FEM)) at the stage of evaluation of the designed structure. The end of the first decade of this century has brought a significant change in the approach to the problem of tissue scaffold design [91]. At that time, the first attempts were made to use optimization algorithms, both classical ones and those based on artificial intelligence methods [92,93,94].

## 5. Biomaterials Used for TE scaffolds 3D Printing

The ideal TE scaffold should be characterized by a number of specific properties, such as adequate mechanical strength and stiffness, open porosity, biocompatibility, as well as biodegradability. Meeting the above requirements makes it possible to create a suitable environment for cell growth. To some extent, all the above-mentioned requirements are due to the material used. Among the materials commonly used for TE scaffolds, one can mention natural (e.g., chitin, collagen, cellulose) and synthetic (e.g., polycaprolactone, polyglycolide, and their copolymers) polymers, as well as ceramics and different kinds of additives (hydroxyapatite (HA), carbon nanotubes). Below, the attempt has been made to characterize the main groups of 3D printable materials. At first, the polymers will be discussed, as the most widely used group of materials for tissue engineering.

### 5.1. Polymers

Polymers represent the main category of materials with high potential for use in 3D printing of TE scaffolds and can be widely used for various tissues’ imitation. TE scaffolds may be fabricated from non-biodegradable as well as biodegradable polymers. In the context of tissue engineering, biodegradable polymers generally have more advantages as compared to the non-biodegradable.

#### 5.1.1. Natural Polymers

Natural polymers are known to be the right candidates for TE scaffold fabrication, mostly due to their bioactivity, biocompatibility, minimal immune response, as well as natural biodegradability of most of them [95]. As an example of the natural polymers’ application in TE, one can mention the work of [96] reporting fabrication of TE scaffolds for cartilage regeneration made of bacterial cellulose. Another study confirms that cellulose from Acetobacter xylinum can be used in the cartilage regeneration [97]. Collagen and chitosan also belong to the polymers widely investigated and applied in TE [98]. All of the above-mentioned materials are known for supporting the cell proliferation and viability [99].

Another biocompatible and easily accessible natural material is gelatine, being an irreversible hydrolyzed form of collagen [100]. There are numerous attempts of using gelatine as biomaterial for 3D printing of TE scaffolds. In the work by [101], the gelatine/hydroxyapatite composite was investigated as material for 3D printing scaffolds for stem cells’ chondrogenic differentiation. Pure gelatine 3D scaffolds were proven to be a good environment for the proliferation and viability of hepatocyte cells [99].

In the work by [102], high proliferation and viability of mesenchymal stem cells cultured on/in collagen/agarose scaffolds wer observed.

#### 5.1.2. Synthetic Polymers

The usefulness of biodegradable synthetic polymers (mainly aliphatic polyesters such as PCL or PLGA) in TE has already been investigated for many years [103,104]. The biodegradable aliphatic polyesters are characterized by relatively low toxicity [105]; however, the acidic oligomeric release, being the effect of polymer hydrolytic degradation, can initiate the inflammatory reaction [106], negatively affecting the tissue regeneration process [107]. Other research works on the degradation kinetics of 3D-printed TE scaffolds made from various aliphatic polyesters, have shown the differences in the degree of the degradation for PLGA (40,000–75,000 Da) and PCL (Mw = 114,000 Da) as 18% and 56% on day 14 and day 28 for PLGA, and 33% on day 21 and 39% on day 28 for PCL, respectively [108]. TE scaffolds made of aliphatic polyesters are known to be successfully applied in the tissue loss treatment [109,110] including bone regeneration [111,112]. The degradation time of TE scaffolds made of aliphatic polymers can be thoroughly controlled [113]. The predominant degradation mechanism for all bioresorbable polyesters used in bioengineering is hydrolysis occurring in enzymatic conditions. From the moment an implant (e.g., TE scaffold) is placed in the living organism, water, which is one of the main components of the physiological environment, penetrates the polymer matrix at various speeds [114]. This penetration speed depends on many factors, including the hydrophilicity of the implant material. Water molecules cause weakening and consequently breaking of ester bonds, which are responsible for the cohesion of polymer chains. It was found that the degradation of some objects made from aliphatic polymers proceeds heterogeneously in such a way that the central part of the object degrades faster than the areas in direct contact with the environment. One can find numerous examples of aliphatic polyesters’ application in tissue engineering [115].

Copolymerization is another way of effectively controlling the final properties of 3D-printed TE scaffolds. Copolymers such as PCL with a PEG (Mn = 1000) addition [116] or PCL (Mw = 2000) with a PLGA addition [117] were synthesized for controlled degradation dedicated to drug-release applications. Different types of printable copolymers, such as poly(hydroxybutyrate) (PHB) [118], poly(propylene fumarate) [119] (PPF), and polyglycolic acid (PGA) [120], were also tested.

Systematic studies concerning bone tissue engineering have been carried out for years. A multitude of 3D-printed scaffolds made of different polyesters and their copolymers were tested under in vivo and in vitro conditions to investigate their abilities for the neo-vascularization and the bone ingrowth [121]. Many works concern the 3D printing of polymeric scaffolds filled with growth factors such as TGF-β and BMP-2 which enable obtaining specifically vascularized bone constructs [122,123].

#### 5.1.3. Hydrogels

Hydrogels belong to crosslinked polymers having the property of binding relatively large amounts of water. They can be made of synthetic or natural polymers such as collagen or alginate [124,125]. Due to their relatively high water content, hydrogels are quite biocompatible and have relatively low mechanical properties. Because of their mechanical similarity to the native tissue, their transport/diffusion properties and high biocompatibility, hydrogels are among the most promising materials from which tissue scaffolds can be fabricated. Moreover, they allow relatively easy and safe immobilization of biologically active molecules. So far, various bioink biomaterials, such as gelatin-methacrylates, agarose, alginate, collagen, chitin, silk, hyaluronic acid, cellulose, and their mixtures have been used together with various crosslinking methods such as click chemistry, ionic/hydrogen bonding, or chemical bonding via radical initiators. Among them, alginates are the most attractive for bioprinting, mainly due to their ability to form a soft gel matrix in a low-aggressive environment for living cells and encapsulated biomolecules. One of the important properties of alginate is its ability to form gels by ionic crosslinking with calcium cations. However, environmental factors such as buffer acidity or temperature can easily affect the condition of the hydrogel material and its degradation, leading to the consequent loss of the biomolecules contained in the hydrogel matrix. Polymers such as poly(ethylene glycol) diacrylate (PEGDA) or natural gelatin methacrylate (GelMA) can also be used for the preparation of hydrogels [126,127]. Hydrogels often are used as a component of hybrid TE scaffolds mimicking the soft tissues (e.g., muscles tissue) [128].

In Table 3, polymer scaffolds with applications and printing methods are summarized.

### 5.2. Other Materials

Ceramic and composite scaffolds contain organic salts of phosphate and calcium. The main advantage of printed 3D ceramic scaffolds is good biocompatibility and very high mechanical strength [176]. Ceramic scaffolds are excellent candidates for bone tissue engineering due to their mineralization ability [177]. Hydroxyapatite (HA), which is a bone component [178], is an attractive material for creating complex 3D structures with mechanical properties similar to those of a bone. These types of 3D-printed scaffolds are widely investigated in regenerative medicine [179]. The above-mentioned ceramic materials can be mixed with a polymer, creating a composite. It was proven that these materials have the ability to support vascularization properties [144,180]. Materials having mechanical properties similar to a bone, such as bioglass, silica, graphene oxide, and zirconium titanate, are often used as the TE scaffold components [181]. The possibility of the fabrication of feasible TE scaffolds made of the polymeric composites containing the mentioned additives was investigated by many groups [182]. Numerous 3D-printed ceramic materials are treated by freeze-drying and sintering to improve cytocompatibility and mechanical properties [183]. TE scaffolds printed from bioactive glass-ceramics with a unique triphasic structure containing hardystonite, gahnite, and strontium were shown to have 34% porosity and a strength similar to that of a bone being 110 MPa [180].

An addition of bioceramics in polymer scaffolds results in excellent properties, higher biocompatibility, and controlled degradation. Furthermore, bioactive ceramics are gaining more and more attention due to their excellent osteogenic properties [184]. Calcium phosphates (CaPs) are the most frequently used bioceramics in tissue engineering applications, due to their similarity to the chemical structure of a bone.

Table 4 summarizes ceramic scaffolds with an addition of a polymer(s) and the printing method used.

## 6. Advanced 3D-Printed TE Constructs—Examples

In this chapter, the selected latest advances in the 3D printing of TE scaffolds are presented, focusing on the new possibilities of the recapitulation of complex tissue structures offered by modern 3D printing techniques.

### 6.1. Nervous Tissue

The central nervous system (CNS) and the peripheral nervous system (PNS) are the most challenging tissues for repair. The 3D printing in vitro model of a brain was developed by forming microchannels with collagen, using needles and a 3D printing frame. Mouse brain cells were cultured on the collagen microchannels, which resulted in regeneration of the brain microvasculature. This experiment has shown that the model of the brain-blood barrier can be used for pathological and physiological tests and many applications, such as drug delivery, tissue regeneration, and tissue engineering [210]. Some studies are devoted to the 3D printing of nerve conduits. In work by [211], cryopolymerized gelatin methacryloyl (cryoGelMA) gel cellularized with adipose-derived stem cells (ASCs) were used for the 3D printing of cellularized conduits for peripheral nerve regeneration. The re-innervation ability of the fabricated conduits was proven in vivo. It is worth mentioning that 3D printing was used for the fabrication of patient-specific casting molds.

### 6.2. Ocular Tissues

Interest in 3D printing techniques in ophthalmology is still growing; however, the majority of 3D printing applications does not concern tissue engineering. Here are examples of works on using 3D printing for ocular tissue regeneration: In the work by [212], an attempt of the reconstruction of a 3D retina is reported. The retina-like structure containing adult rat retinal ganglion cells and glia were 3D printed. It was proven that these types of retinal cells can be successfully printed without loss of viability and certain phenotypic features. Another example of the application of 3D printing in ocular tissue engineering would be the work by [213] concerning the fabrication of the TE corneal scaffold made of collagen-based bio-ink containing encapsulated corneal keratocytes.

### 6.3. Ear

The computer-aided design has been used to create the bionic human ear. A hydrogel matrix containing cells and a conductive polymer with the addition of silver nanoparticles were used during printing—bioprinted in the shape of a human ear. The studies allowed control of the signals from the cochlea-shaped electrodes. The in vitro culture was provided on the cartilage tissues on every side of the inductive coil. The printed ear was found to enhance the auditory sensing. Another study showed that the printed ear can be formed by 3D bioprinting with the subject’s lipid tissue and an auricular cartilage. Adipocytes and chondrocytes differentiated from the adipose-derived stromal cells were enclosed in hydrogels and then placed at the lipid and cartilage tissue [214,215,216].

### 6.4. Kidney

Scaffolds from PEGDA with the addition of sodium alginate and calcium sulfate were tested [150]. After fabrication, scaffolds were crosslinked using UVclight, and subsequent human embryonic kidney cells (HEK) were cultured. It was shown that the mentioned composite materials are characterized by properties supporting the proliferation and viability of the cells. In the work of Lawlor et al. [217], extrusion-based 3D bioprinting was applied for the generation of human kidney organoids (the organoid is a simplified version of a living organ produced in vitro). The used fabrication method enables for precise manipulation of organoid size and cell number and conformation. The developed in vitro model of kidney organoids could be used for drug testing or disease modeling.

### 6.5. Skin

Using a laser-assisted method, a 3D-printed skin was developed. Collagen type I and Matriderm (for matrix stabilization) were mixed and cultured with fibroblasts and keratinocytes. The experiment was also performed at in-vivo conditions by placing a bioprinted construct on the murine skin. In the effect, mainly an epidermis forming was observed [218]. In [219], the method of biofabrication of skin equivalents (SE) that are bioprinted using open-market bioprinter, made with fibroblasts and keratinocytes suspended in the gelatin-based hydrogel, was discussed. SE construct layers were extruded directly onto the multi-well plate. Three levels comprise the developed structure: dermis, laminin/entactin basal layer, and epidermis. The developed SE may be used for in vitro skin disease modeling.

### 6.6. Cancer Models

Recent progress in bioprinting enables the development of 3D in vitro models of various kinds of cancerous tissue [220]. Such models enable the design of patient-specific therapies as well as for the investigation of the processes related to carcinogenesis, such as tumor extravasation [221]. Bioprinted cancer models usually are composed of multiple layers containing different cell types including tumor cells (usually patient-derived cells), the extracellular matrix, growth factors, and vasculature [222]. Bioprinted tumor models should recapitulate the actual tumor heterogeneity. They enable anti-cancer therapy screening as well as the investigation of cell-cell and cell-matrix interactions. Bioprinted cancer models are characterized by great advantages over 2D in vitro models, which cannot mimic the structural complexity of tumors.

### 6.7. Bone and Cartilage Tissue Engineering

Bone and cartilage defects repair is one of the most common regenerative procedures. The principal part of bone and cartilage tissue engineering is to replace a damaged bone. Therefore, 3D printing techniques try to print a structure of artificial bone with required properties, such as appropriate mechanical properties, shape, and size [223]. The major causes of bone and cartilage defects are trauma, congenital anomalies, and tissue resection due to cancer. Such treatments such as autogenous bone grafting are characterized by several disadvantages, such as unsuitable donor tissue availability or donor site morbidity. On the other hand, allogeneic bone grafts are avoided mainly due to the risk of disease transmission. Over the past several years, the importance of therapies using the 3D-printed TE scaffolds has been growing gradually. TE scaffolds enable seeded cells to adhere, migrate, grow, and differentiate into chondrogenesis and osteogenesis.

Here are examples of recently published works on the application of 3D printing in bone and cartilage regeneration: Most of the proposed solutions are based on the combination of several different materials—ceramic, polyesters, and hydrogels [224,225,226] Quite often, to improve the cell-seeding efficiency and osteoinductivity, an injectable hydrogel is incorporated into a 3D-printed porous structure to form a hybrid scaffold [227]. Despite the fact that multiple types of materials are used to fabricate 3D-printed bone scaffolds, biodegradable aliphatic polyesters remain the gold standard [228,229]. On the other hand, hydrogels are the most popular group of materials for the cartilage TE [230]. Osteochondral scaffolds remain a particular challenge for tissue engineering. Typically, the fabrication of osteochondral scaffolds requires a combination of several printing techniques and materials [231], as it should be remembered that osteochondral scaffolds are usually bi- or even tri-phasic.

## 7. Future Directions and Conclusions

Various approaches in scaffolds’ formation for use in tissue engineering applications are experiencing rapid advances. Regarding the development of 3D-printed scaffolds, the most important goal is to mimic the complexity of a natural living tissue truly. Its structure should have appropriate mechanical properties, pore size distribution, and pores’ arrangement (allowing cell migration and diffusion). While numerous tissues were successfully cultured as proof of the principle, the development of a fully functional complex human-size organ is still pending.

The types of fabrication methods and the materials provided in this review serve to improve current TE procedures.

### Drawbacks and Future Directions of the 3D Printing of TE Scaffolds

Even though 3D printing is extremely promising from a TE point of view, it is characterized by several limitations related mainly to the lack of legal regulations and standardized procedures. Moreover, the fabrication of any TE product requires advanced and costly infrastructure that may include software, robust computer workstations, 3D printers, and cell culture laboratory facilities. Nowadays, most TE product implantation attempts were realized in cooperation between hospitals and research institutes. Usually, such cooperation was of an ad hoc nature and did not go beyond the research study. It is clear that to increase the availability of 3D printing in TE applications, the current collaboration model between engineers and doctors needs to be modified. One of the ideas is to establish regional 3D printing centers, adequately equipped and staffed [232]. Such centers would contribute to more efficient use of the equipment and human resources. The idea is that such centers could serve many medical facilities in a given region.

A major problem facing researchers, doctors, and engineers is the lack of an established legal framework and procedures for validating tissue-engineered products. It is a well-known fact that existing legal provisions hinder the scale-up from the laboratory to a larger scale. Attesting of patient-specific TE products is also problematic from the point of view of current standards.

Another drawback related to the 3D printing of TE scaffolds is a lack of standardized terminology used to systematize the field, which is a characteristic of new and rapidly developing fields of knowledge.

Still, the actual issue and the most challenging step involve a translation of the technology to the next level—the availability to the patients, giving a chance to improve the quality of their lives. Not so long ago, the 3D-printing-assisted cultivation of TE constructs has been started using the patient’s cells [233,234], and now the symbol of the cutting-edge technology for TE is 4D printing [235]. This advanced 3D-printed technology adds a fourth dimension—time—to currently-used 3D printing. It enables the fabrication of TE scaffolds having a self-assembly ability. This technique assumes the use of smart materials characterized by the ability to change their properties under the influence of an applied stimulus (e.g., thermoresponsive shape memory polymers). Furthermore, 4D printing can be used for the fabrication of TE scaffolds enabling the mechanical stimulation of living cells by the external signal (e.g., magnetic field) [236]. Vascularization is another challenging goal, and the new generation of bioprinters with multiple print-heads seems very promising. Loaded with various cell types, they are expected to reconstruct and recapitulate the factual complexity of a multi-tissue organ.

Future activities should include testing materials for medical-oriented 3D printing methods, creating new printers to provide high precision of TE scaffolds, making unified standards for scaffolds, strengthening market supervision to optimize implants for clinical use, and establishing a 3D printing platform to enhance communication among research institutes, hospitals, and companies. These advancements should further promote the development of 3D-printed tissue engineering technology.

## Figures and Tables

**Table 1:** Selected scaffolds’ formation techniques—main applications and advantages/disadvantages.

Method	Applications	Advantages	Disadvantages
Electrospinning	Bone, nerve, skin, and cardiac tissue engineering [28]	High surface area to volume ratio, high porosity, easy process	Limited range of polymers
Phase separation	Protein delivery applications and/or drug release [29]	Bioactive agents can be incorporated into the structure, high porosity	Limited ranges of pore size, problems with residual solvents
Solvent casting	Vascular tissue engineering applications [30]	Simple method, controlled porosity	Low mechanical strength, limited thickness, small pore size

**Table 2:** The most popular 3D TE Scaffolds fabrication techniques—applications, advantages, and disadvantages.

Method	Applications	Advantages	Disadvantages
Bioprinting	- scaffolds manufacturing [71] - hydrogels [72] - tissue engineering [73,74] - cell growth [75]	- prints viable cells - soft tissue applications	- requires support structures - nozzle limitations - must be not cytotoxic during process
Extrusion-based methods	pharmaceuticals [76,77] scaffold manufacturing [78] bone tissue engineering [79] cardiovascular medical devices [80]	- low cytotoxicity - low cost [81] - inexpensive printers	- only thermoplastics materials [82] - low resolution [81] - non-biodegradable materials can be used - post-processing
Indirect methods (Selective Laser Sintering; Stereolitography)	- pharmaceutical [83] - biomedical manufacturing [84,85] - bone tissue engineering [86,87] - pharmaceutical [76] - drug delivery [88]	- high mechanical properties - SLS: powder supporting the structure - high resolution - smooth surface - short time of the process	- photo-sensitive materials - expensive - support systems in case of very complicated structures

**Table 3:** Polymer scaffolds with applications and printing methods.

Polymer Scaffolds	Printing Method	Applications	Refs.
Chitosan/Rhizopus mycelia/Fungi	-	Bone regeneration	[129]
PCL	Direct Printing	Heart and cartilage tissue	[130]
PCL	FDM	Tissue engineering	[131]
PCL/alginate-based hydrogel	Extrusion	Bone tissue engineering	[132]
PCL/PLA	Bioextrusion	Tissue engineering	[133]
PCL, chitosan	FDM	Bone tissue engineering	[134]
PCL/HA	FDM	Tissue engineering	[135]
PCL/silk	Extrusion	Tissue engineering	[136]
PCL/castor oil	FDM	Bone tissue engineering	[137]
PCL	FDM	Bone tissue engineering	[138]
PCL/HA	Indirect printing	Tissue engineering	[139]
PCL/diamond	Extrusion	Tissue engineering	[140]
PLA, PLGA, collagen	FDM	Tendon-bone	[141]
PLA, collagen	FDM	Bone tissue engineering	[142]
PLA	FDM	Bone tissue engineering	[143]
PLCL	FDM	Tissue engineering	[144]
PLA/ABS	FDM	Bone tissue engineering	[145]
PLA	FDM	Bone tissue engineering	[146]
PLA/cellulose	Extrusion	Tissue engineering	[147]
PCL, PLGA, collagen, gelatin	FDM, extrusion	Bone tissue engineering	[148]
PLCL/dECM	Hot melting Extrusion	Tissue regeneration	[149]
Alginate, PEGDA, CS	Extrusion	Kidney	[150]
Alginate	Extrusion	Microphysiologic studies	[148,151]
Alginate, collagen, agarose	Extrusion	Cartilage	[152]
Alginate, gelatin	Extrusion	Mutlicellular tissue	[153]
GelMA/Alg-PEG-M	Extrusion	Vascular	[154]
Agarose, collagen	Extrusion	Kidney	[102]
PCL	3D printing	HOb	[155]
PC	3D printing	Bone tissue engineering	[156]
Me-HA/GelMA	Extrusion	Cardiac tissues	[157]
Me-HA	Extrusion	Bone tissue engineering	[158]
Agarose/carbon nanotubes	Extrusion	Biosensors, various tissues	[159]
PVA, phytagel	Extrusion	Soft connective tissue	[160]
Gelatin/silk fibroin	Extrusion	Skin	[161]
Hyaluronic acide/gelatin	Extrusion	Cardiac	[162]
Collagen/chitosan	Extrusion	Neural tissue engineering	[163]
Alginate/gelatin	Extrusion	Tumor microenvironment	[164]
Pluronics/gelatin methacrylate	Extrusion	Vascular	[165]
Alginate	Extrusion	Liver	[166]
NFC, alginate, hyaluronic acid	Extrusion	Cartilage	[167]
NFC/alginate	Extrusion	Cartilage	[168]
Collagen	Extrusion	Skin	[169]
Porcine skin powder	Bioprinting	Soft tissue engineering	[170]
HA, PLGA	Stereolithography	Bone tissue engineering	[171]
PLA/PCL/HA	Extrusion	Cartilage defects treatment	[172]
PEGDA, polydiacetylene nanoparticles	Stereolithography	Liver tissues	[173]
VE/VC	DLP	Bone tissue engineering	[174]
Cellulose nanocrystal	DIW	Multicellular tissue	[175]
PLGA	Inkjet	Liver tissues	[115]

PCL—polycaprolactone; PLA—polylactic acid; HA—hydroxyapatite; PLGA—poly Lactic-co-glycolic acid; PLCL—Polyl-lactide-co-ε-caprolactone; ABS—acrylonitrile butadiene styrene; PEGDA—poly(ethylene glycol) diacrylate; CS—cellularized structures; Me-HA—methacrylated hyaluronic acid; GelMA—metharylated gelatin; Alg-PEG-M—alginate, poly ethylene glycol tetra acrylate; PC—polycarbonate; PVA—polyvinyl alcohol; NFC—nanofibrillated cellulose; VE—vinylester, VC—vinylcarbonate.

**Table 4:** Ceramic scaffolds with/without an addition of a polymer(s) and the printing method.

Ceramics	Polymer(s)	Printing Method	Refs.
BCP	PCL	Inkjet	[185]
HA/TCP	-	digital light processing (DLP)-type 3D printing system	[186]
BCP	PLGA, PCL, collagen	FDM	[187]
β-TCP	PEGDA	Stereolithography	[188]
zirconia polycrystal (3Y-TZP) and Pluronic hydrogel ceramic paste	Bisphenol A glycerolate dimethacrylate (Bis-GMA) and tri(ethylenglycol) dimethacrylate (TEGDMA) copolymer	3D-printed by robocasting method	[189]
HA	PLA	FDM	[190,191]
HA	PCL	FDM	[192]
HA, bone marrow clots	PCL	FDM	[193]
HA, PLGA microspheres	PCL	FDM	[194]
HA, solvent system	PLGA	Extrusion	[195]
HA, α-TCP, phosphoric acid	Collagen	Inkjet	[196]
Ti6Al4V		Laser beam melting	[197]
Titanium	PLA	3D printing based on Fused Filament Fabrication (FFF)	[198]
Mesoporous silica, CPC		Extrusion	[199]
Titanium, platelets	Gelatin	Laser sintering	[200]
CPC		Extrusion	[201]
Calcium silicate	PCL	Laser sintering	[202]
Mesoporous bioglass, CS		Extrusion	[203]
Wallastonite, magnesium		Extrusion	[204]
BCP, HPMC, ZrO2		Extrusion	[205]
CS		Inkjet	[206]
Silica, calcium carbonate		Laser assisted gelling	[207]
Tricalcium phosphate		Inkjet	[208]
Graphene	PCL	FDM	[209]

BCP—tricalcium phosphate-hydroxyapatite bioceramic; PCL—polycaprolactone; HA—hydroxyapatite; TCP—tricalcium phosphate; PLGA—poly Lactic-co-glycolic acid; PEGDA—poly(ethylene glycol) diacrylate; CPC—calcium phosphate cement; HPMC—Hydroxypropyl methylcellulose; CS—cellularized structures.

## Data Availability

No new data were created or analyzed in this study.
